# Valsalva Leading to Pain in the Left Arm: A Case of Paradoxical Embolism

**DOI:** 10.7759/cureus.80834

**Published:** 2025-03-19

**Authors:** Sami Refai, Ammar Mattar, Zuka Safarjalani, Asad Syed, Rana Haggag, Richard Virgilio

**Affiliations:** 1 Internal Medicine, Edward Via College of Osteopathic Medicine, Auburn, USA; 2 Clinical Affairs, Edward Via College of Osteopathic Medicine, Auburn, USA

**Keywords:** acute upper limb ischemia, arterial emboli, paradoxical embolism, pfo occluder device, ultrasound-guided

## Abstract

Paradoxical embolism (PDE) secondary to a patent foramen ovale (PFO) is an underdiagnosed condition with potentially severe clinical outcomes, including stroke, myocardial infarction, migraines, and peripheral arterial embolism. PFO is typically asymptomatic and often requires a high index of suspicion for a proper diagnosis. We report a case of a 36-year-old male smoker with a medical history significant for pulmonary thromboembolism, deep vein thrombosis (DVT) of the right lower extremity (RLE), type 1 diabetes mellitus (T1DM), and coronary artery disease (CAD).

The patient presented with sudden-onset left arm pain after recreational activity. Physical examination revealed a hemodynamically stable individual with findings suggestive of ischemia in the fourth and fifth digits of the left hand. Initial diagnostic workup including duplex ultrasound, CT angiography (CTA), and transthoracic echocardiography (TTE) suggested left ulnar artery thrombosis, with TTE negative for cardiac septal abnormalities. Following the thrombectomy, he experienced complete symptom resolution upon discharge. However, two weeks later, he returned with identical symptoms and clinical findings. Subsequent investigations, including transesophageal echocardiography (TEE) with a bubble study, confirmed the presence of a PFO. The recurrent thrombotic event was attributed to a PDE facilitated by the PFO. Definitive management involved PFO closure using a septal occluder device, resulting in the successful resolution of symptoms. This report highlights the importance of considering PFO in patients with unexplained recurrent embolic events and underscores the value of advanced imaging modalities, such as TEE, in establishing the diagnosis.

## Introduction

Paradoxical embolism (PDE) occurs when an embolus circumvents pulmonary circulation via a patent foramen ovale (PFO) and enters systemic circulation [1]. While it is estimated that about 25% of the world’s adult population experiences a PFO, many are asymptomatic [2]. Obstructive events due to a PDE most commonly manifest as a cerebrovascular accident while acute limb ischemia is much rarer [3]. Repeated episodes of acute limb ischemia in the same artery should raise suspicion for more infrequent causes for the occlusive event. Acknowledging this as a potential etiology is crucial for guiding diagnostic evaluation and treatment. We discuss a case of a young adult male who experienced two occlusive events in the same arterial site after performing a Valsalva-like event while playing with his children, likely due to PDE.

## Case presentation

The patient was a 36-year-old obese (BMI: 33 kg/m^2^) male with a past medical history notable for a right lower extremity (RLE) deep vein thrombosis (DVT) despite earlier thrombectomy two months prior and was on warfarin, suspected of nonadherence. Other notable medical history included pulmonary embolism (PE), coronary artery disease (CAD) myocardial infarction (MI) status post percutaneous coronary intervention (s/p PCI), type 1 diabetes mellitus (T1DM), and a family history of factor V Leiden mutation. The patient presented to a vascular clinic with pain, numbness, and discoloration in the left upper extremity (LUE) after reaching to grab something from across the pool without falling in. The patient described left arm numbness that felt like "rubber". He also admitted to having temporary weakness in his left arm, but he soon regained function, with residual numbness in the fifth and part of the fourth digits of the LUE.

**Figure 1:**
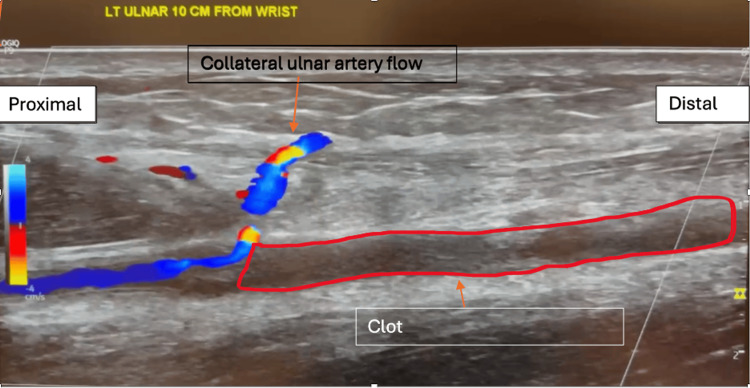
Duplex ultrasound image depicting the patient's ulnar artery clot on first admission to the emergency department

The patient denied having chest pain, shortness of breath, or other symptoms. The vascular outpatient clinic performed an initial workup of the patient, including an EKG and color duplex ultrasound (US) as seen in Figure 1. EKG was normal; however, the US revealed left ulnar arterial occlusion, prompting the physician at the vascular clinic to recommend that the patient visit the emergency department (ED).

An initial evaluation was performed at the ED. The patient's physical exam raised concerns for ischemia of the distal tip of the second digit along with ischemia of the fourth and fifth digits of his left hand. He was started on rivaroxaban in the ED, and vascular surgery was consulted. Vascular surgery requested a cardiac workup, including troponin, EKG, and CT angiogram (CTA) chest with LUE runoff. Troponin and EKG were normal. Vascular surgery admitted the patient to the medical/surgical floor and switched him from oral rivaroxaban to heparin intravenous (IV) infusion.

CTA revealed a small right-sided sub-segmental PE and an occlusion of the left ulnar artery roughly halfway down the forearm approximately 7 cm from the brachial artery bifurcation. The mid/distal ulnar artery was completely thrombosed, while the remainder of the left upper extremity arteries were normal. Patient history revealed that his most recent thrombectomy of the DVT had been two months prior. The patient also had a known family history of factor V Leiden mutation but was negative for this genetic defect upon further testing. A transthoracic echocardiogram (TTE) was performed at the time of admission, which originally did not reveal the presence of any atrial abnormalities such as a PFO. A thrombectomy performed on admission day two revealed multiple cylindrical-shaped segments of reddish-brown hemorrhagic-appearing soft tissue consistent with thrombi. The patient was discharged on admission day three and was prescribed warfarin with a temporary course of enoxaparin to provide anticoagulation until his warfarin reached the appropriate therapeutic range.

Eleven days later, the patient presented to the ED again for a recurrence of arterial occlusion. He was initially evaluated at the same outpatient vascular clinic where they performed duplex US. The vascular physician recommended visiting the ED after discovering that an ulnar arterial occlusion had reoccurred in the left arm. The patient was tachycardic but hemodynamically stable upon arrival at the ED. On exam, he had an area of old-appearing ecchymosis to the left anterior forearm and 2+ radial pulse with good distal perfusion. His left ulnar artery thrombectomy surgical site had no signs of infection. Muscle strength testing showed 4/5 strength with flexion of the fourth and fifth digits of the left hand, as well as subjective numbness. He had no sign of acute limb ischemia. Clinical presentation resembled his initial visit to the ED, with pain, numbness, and discoloration in the LUE being most prominent. The patient also reported twitching in his fourth and fifth digits. He still had the right DVT managed with warfarin and enoxaparin. Warfarin and enoxaparin were discontinued during the admission and the patient was started on heparin IV infusion and admitted to the medical/surgical floor, where they ordered arterial ultrasound, CTA, and coagulation studies.

**Figure 2:**
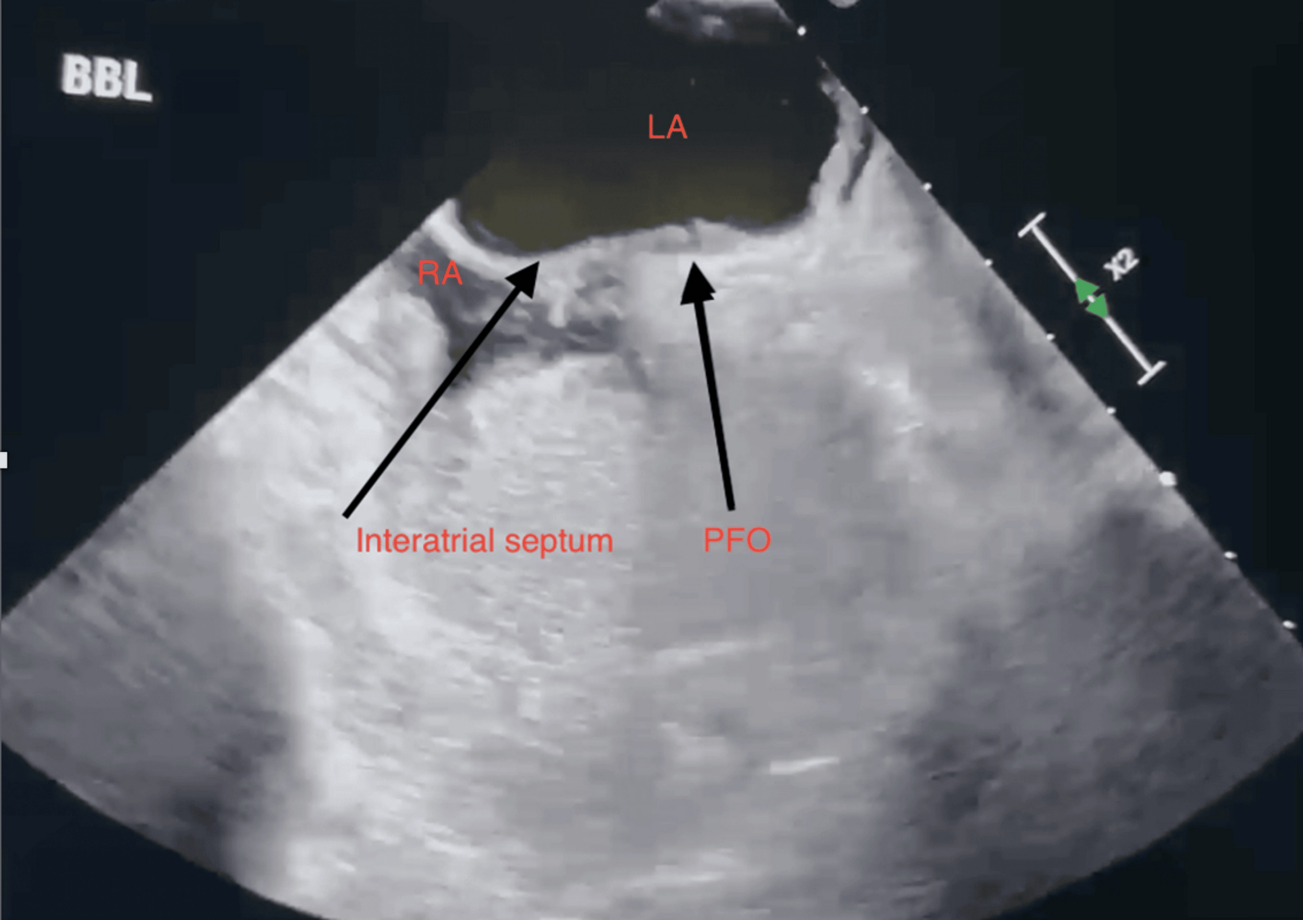
Transesophageal echocardiogram long-axis view displaying structures surrounding PFO LA: left atrium; PFO: patent foramen ovale; RA: right atrium

Labs and a repeat ultrasound showed an international normalized ratio (INR) of 1.7 (target therapeutic range: INR 2-3) and a recurrence of his left ulnar artery occlusion. The following day, labs revealed hemoglobin of 13.3 g/dL (normal range: 13.5-17.0), prothrombin time (PT) of 23.3 seconds (normal range: 12-14.5), partial thromboplastin time (PTT) of 97 seconds (normal range: 25-35), and an improvement in INR at 2.06. CTA revealed a complete occlusion of the mid/distal left ulnar artery approximately 7 cm from the brachial artery bifurcation. The mid/distal ulnar artery was thrombosed entirely. The intraosseous artery was patent, with small caliber opacified up to the mid-forearm. The remainder of the LUE arteries were normal. Transesophageal echocardiography (TEE) with a bubble test was ordered. TTE revealed a PFO as seen in Figure 2.

Successful intracardiac echo-guided PFO closure with a 30 mm GORE Septal Occluder device (W. L. Gore & Associates, Inc., Flagstaff, AZ) was carried out on admission day three with no complications. A chest x-ray confirmed the placement of the device. After his stable recovery, the patient was discharged on admission day five with regular INR checks, a one-month follow-up with repeat TTE, and education on warfarin management. Chest X-ray confirmed the placement and closure of the PFO. As for the ulnar arterial clot, vascular surgery decided to not remove it. An aspirin and statin regimen was added to the patient’s medication plan given his CAD.

## Discussion

This report highlights the challenge of diagnosing PDE due to PFO and the importance of ruling out alternative thrombotic and embolic causes. Our patient's presentation with acute left upper limb ischemia led to a systematic evaluation of potential etiologies, ultimately supporting PDE as the most plausible diagnosis. Workup for a PFO-mediated PDE should only be performed once other more likely causes of arterial embolism have been ruled out [4]

Differential diagnosis considerations

PFO-related PDEs are implicated in 87% of ischemic strokes with no identifiable cause [5,6] and are also associated with myocardial infarctions, migraines, and acute peripheral thromboembolic occlusion [4,7,8]. While limb ischemia is more prevalent in the lower extremities [9], acute upper limb ischemia (AULI) is relatively rare, accounting for 10-15% of all acute limb ischemia cases [10]. The most common embolic source of AULI is cardiac, particularly atrial fibrillation (AF) [11]. The brachial artery is the primary site of embolus lodgment [11], and the relative risk of thromboembolectomy for AF patients is 7.5 for men and 9.3 for women [11]. Other significant risk factors include hypertension, myocardial infarction, heart failure, large aortic plaques (>5 mm), intracardiac thrombi, aneurysms, and hypercoagulable states [11].

An acute arterial occlusion can result from two primary mechanisms: in-situ thrombosis or embolism. Thrombus formation may occur due to vessel trauma, hypercoagulable status, or hypertension [12]. Primary hypercoagulable conditions, including lupus anticoagulant, anticardiolipin antibodies, factor V Leiden and thrombin gene mutation, heparin-induced thrombocytopenia thrombosis, and other rare causes significantly increase thrombotic risk [12]. Secondary hypercoagulable states, such as malignancy, myeloproliferative disorders diabetes mellitus, trauma, iatrogenic injection, and smoking, further exacerbate thrombus formation risk [12]. Despite negative laboratory results for the aforementioned hypercoagulability risks, and no evidence of unexplained weight loss to indicate malignancy, our patient still had multiple thrombotic risk factors, including T1DM, obesity, CAD, prior MI, prior pulmonary thromboembolism (PTE), chronic right femoral DVT, and subtherapeutic INR. While these factors collectively increased the likelihood of thrombosis, they alone could not fully explain the pathophysiology of the patient's acute arterial occlusion.

Thrombi typically develops gradually, with symptoms progressing insidiously [12]. The severity of an acute arterial occlusion depends on the extent of collateral circulation [12]. If sufficient collateral circulation is present, symptoms may be minimal or absent, often seen when an acute thrombus forms on pre-existing chronic stenosis [12]. Atherosclerotic plaques allow time for collateral formation, mitigating ischemic symptoms [12]. The upper extremity possesses greater collateral circulation than other vascular territories, which generally reduces the incidence of thrombotic occlusions [13]. However, isolated thrombotic occlusions in the upper limb remain rare. Given this patient's history and the sudden onset of symptoms, an embolic etiology was considered more likely than in-situ thrombosis.

Embolic etiology

Arterial embolism often presents with acute onset pain, numbness, tingling, and discoloration of the affected limb [12]. Embolism to the upper extremity can arise from both cardiac and non-cardiac sources. Cardiac emboli typically originate from atrial fibrillation, prosthetic valves, or ventricular aneurysms, whereas non-cardiac embolic sources include aortic plaques, ascending aortic aneurysms, subclavian artery aneurysms from thoracic outlet syndrome, and subclavian artery stenosis [12]. Angiography remains the standard for evaluating distal arterial occlusions [12]. In this patient, CTA of the chest and left upper extremity demonstrated complete patency of all major vessels except for the left ulnar artery. Without clear evidence of a thrombotic or embolic source in the left heart or proximal upper extremity, these common sources of embolism were effectively ruled out based on diagnostic testing. Additionally, this patient’s EKG did not reveal the absence of P-waves to indicate atrial fibrillation, although, paroxysmal atrial fibrillation could not be entirely ruled out.

Paradoxical embolism as the leading diagnosis

Given the exclusion of traditional embolic causes such as AF or ventricular aneurysms, the probability of an occlusion due to a PDE increases. When his pre-existing risk factors are considered such as long-standing right femoral DVT, subtherapeutic INR, and the onset of symptoms after a Valsalva-like maneuver, the diagnosis is further supported. PDE occurs when a venous thrombus bypasses the pulmonary circulation via a right-to-left shunt, such as a PFO, leading to arterial embolization. Normally, the left atrial pressure exceeds that of the right atrium, keeping the atrial septa closely apposed and preventing any right-to-left shunting. Valsalva maneuvers and increased abdominal pressure have been shown to significantly increase right atrial pressure which is likely how the embolus skipped pulmonary circulation [14].

Patent foramen ovale can be detected by several different imaging modalities, with the gold standard method being a TEE [15]. When the patient first presented with an occlusive event, TTE with bubble study and Valsalva did not identify a PFO. TTE with bubble study and Valsalva was initially preferred due to its non-invasiveness and avoidance of anesthesia. However, a TTE is inferior to a TEE, and it was noted that the test was limited due to patient movement.

Management and outcomes

Guidelines for the treatment of PDE due to a PFO are not well established. Any form of acute limb ischemia requires prompt treatment and a focus on restoring perfusion and limb salvage [3]. Surgical embolectomy/thrombectomy of an arterial occlusion is the preferred method of treatment, other options may include fibrinolysis, as well as prompt anticoagulation pre- and post-operation [3]. Thrombogenic or embolic causes of arterial occlusions in cardiac or non-cardiac areas of the body must be explored.

During the first incident, the patient received IV heparin to prevent further clot propagation, followed by a thrombectomy to restore perfusion. On Doppler insonation, the ulnar artery had poor diastolic flow compared to the patient's palmar arch signal There were no deficiencies post-op, and the patient was discharged with systemic anticoagulation in accordance with clinical guidelines [16]. In the literature, immediate anticoagulation was initiated in most cases and attempts to remove the causative embolus were undertaken in most instances [3].

The recurrence of arterial occlusion prompted a PFO closure to prevent future embolic events. The PFO was successfully sealed using intracardiac echo-guided (ICE) closure via a 30 mm GORE Septal Occluder device under conscious sedation. The closure was confirmed via chest X-ray to confirm the placement of the device and TTE with a "bubbly" and Valsalva to confirm a complete seal. The second clot was not removed at the discretion of vascular surgery and did not believe that removing the embolus would benefit the longevity of the patient's ulnar artery. Post-procedural follow-up showed no recurrent thrombotic events. Five months later, the patient followed up with an outpatient vascular physician with no symptoms and full functionality restored.

Individuals who experience any form of peripheral vascular disease must make significant lifestyle adjustments [17]. Before discharge, the patient was extensively counseled on smoking cessation. Tobacco smoke induces oxidative stress, and nicotine can propagate plaque formation [17]. Decreasing lipids and tighter control of blood glucose levels will help slow the progression of atherosclerotic plaques, especially since this individual has a past medical history of MI.

Limitations

The diagnosis of a PDE is generally a diagnosis of exclusion and is scrutinized in the literature. Patients with suspected PDE tend to have multiple confounding factors for thromboembolic events [18]. Although this case outlines various evidence for a PDE, this patient’s history may reveal a different pathophysiology behind this arterial occlusion. One such factor includes the patient's subtherapeutic INR, which may have increased the likelihood of random thrombotic events in both the venous and arterial circulation. A meta-analysis assessing INR control in VTE patients found that poor INR control was associated with a higher risk of recurrent VTE [19]. Additionally, one in five thromboembolic events occurred during periods of low INR, highlighting the role of subtherapeutic anticoagulation in occlusive events [20].

INR monitoring with a target range between 2-3 was discussed extensively with this patient. Nonadherence to INR monitoring was associated with a higher risk of thromboembolism [21]. In summary, subtherapeutic INR levels are associated with an increased risk of arterial occlusion. Hence, maintaining therapeutic INR levels is essential to reduce this risk.

## Conclusions

PDE should be considered in patients presenting with unexplained ischemia, particularly in the setting of known thrombotic risk factors. A confirmed PFO, chronic history of venous thrombosis, and no recurrent arterial occlusion following PFO occlusion support a diagnosis of a PDE of the ulnar artery. This report reinforces the importance of a thorough diagnostic evaluation and the potential benefits of PFO closure in preventing further complications in patients with recurrent embolic events.
